# Clinical Remarks on the Value of Belladonna in the Treatment of Iritis

**Published:** 1865-03

**Authors:** Jabez Hogg

**Affiliations:** Assistant-Surgeon Royal Westminster Ophthalmic Hospital, &c.


### CLINICAL REMARKS ON THE VALUE OF BELLADONNA IN THE TREATMENT OF IRITIS.

By JABEZ HOGG, Esq., Assistant-Surgeon Royal Westminster Ophthalmic Hospital; Editor of the Journal of British Ophthalmology.

After a careful examination into the relative value of the various agents for the cure of iritis, and opium in particular, I believe I am perfectly justified in saying that the chief value of the latter consists in its sedative or narcotic principle, when employed in sufficient doses, for the relief of the pain attendant upon the attack. If we take the trouble to examine a little closely into the published cases of alleged cures without mercury, or with opium, we shall find, remarkably enough, that in nearly all, at some period of the treatment another remedy, belladonna, has been employed, perhaps applied externally, in combination with some other potent drug. For example—on looking over the cases published by Dr. Williams, of Boston, we at once notice the secret of his success lies in this practice—he invariably commences the treatment "with an *instillation of a solution of atropine;*" this is ordered to be repeated until complete dilation of the pupil is produced; at the same time iodide of potassium or some other equally efficacious remedy is prescribed in full doses. What better treatment in the majority of cases could be adopted? There are other practitioners, we fear, who, while professing to cure by the agency of morphia alone, do not possess the honesty of Dr. Williams, but are most careful to conceal what they employ in conjunction with their boasted remedy.

It is generally conceded that in all cases of iritis, the greatest possible danger to be apprehended is in the formation of adhesions between the iris and the capsule of the lens, posterior synechia, or between it and the cornea, anterior synechia; the latter is not of very frequent occurrence. In the acute stage of the attack lymph is effused, and the pupillary space wholly or partially obliterated in a comparatively short time, and thus vision will be quickly lost, even should the choroid and retina escape the destructive process set up by the iritis. In milder attacks the danger of synechia and a fixed pupil cannot always be avoided, more especially if the attack is unheeded by the patient, or if not seen by a medical man during the first thirty or forty hours. Even if promptly attended to, the patient may not escape with a perfect iris; and therefore the case can only be said to be cured if no adhesions or irregularity of the pupil remain after the attack has been entirely subdued.

It is quite obvious, indeed, that our chief care in the treatment of this malady must be directed to preserve the iris intact, free from the smallest deposit, and quite circular. To fulfil this indication we must, from the very first, keep the pupil well dilated, and thus place it as much as possible out of harm's way and in a state of rest. To expect to effect this by any so-called constitutional remedies, without at the same time employing belladonna or atropine, is to neglect a most important point of treatment and jeopardize your patient's vision. Whereas, by a liberal use of belladonna or a solution of atropine (2 to 4 grs. to the oz.), or a fiftieth part of a grain of a very finely levigated powder of the sulphate of atropine, applied to the conjunctiva of the lower eyelid, and repeated at intervals of three or four hours, until the pupil becomes fully dilated, and keeping it in this condition, we may, I believe, almost adopt any kind of treatment, or even leave the patient to careful nursing, particularly if the case be one of a non-specific uncomplicated nature. After the pupil has been fully dilated, the further application of atropine once daily is quite sufficient to maintain the full effect and keep the iris free from danger; but if the lymph or interstitial deposits can already be detected, it will be found to be much more difficult to dilate the pupil, and it is under these circumstances that our good old remedies, mercury, iodide of potassium, &c., come into play with the happiest results, and will not only preserve sight, but save the iris from complete disorganization.

In England, the value of mydriatics in the treatment of iritis has been very generally recognized, and always highly esteemed by our ophthalmic surgeons from a very early period. This is not the case in Germany, and some other countries. Dr. Mackenzie, in his early writings, says:—"Belladonna ought to be employed in every case, and in all stages of the disease. The usual mode of employing it is in extract, moistened to the consistence of cream,* and liberally painted on the brow and eyelids morning and evening. As it is during the night that the disease appears to make most progress, and as during sleep there is a natural closure of the pupil, which must favor the permanent contraction which iritis tends to produce, the evening is evidently the most important time to apply belladonna. After bloodletting it will be found to act much more powerfully in dilating the pupil.

"As soon, in general, as the inflammation has subsided in any considerable degree, and the fibres of the iris have become somewhat freed from effused lymph, the pupil will begin to expand, and even in neglected cases, where it has been allowed to become almost obliterated, the continued use of belladonna for months is sometimes attended by a gradual dilatic[illegible] of the pupil, an elongation of the threads that bind it to the capsule, and a corresponding improvement in vision. After the acute inflammation is gone (subdued by extracting blood, &c.), a filtered aqueous solution of the extract of belladonna, or a solution of the sulphate of atropia, may be dropped into the eye morning and evening; applied thus to the conjunctiva, belladonna has more effect than when painted on the skin, and sometimes breaks through adhesions when smearing the outside of the eyelids has failed."

So little appears to have been known in Germany of the employment of mydriatics, that up to a very recent period we find it stated of a Prussian quack oculist, that he derived a large annual income from patients who literally flocked to him for the cure of cataract, &c. His treatment chiefly consisted in the application of belladonna drops to the eye, belladonna plasters to temples, and mercurials. He kept his secret by supplying his patients with drugs from his own store. It was, therefore, high time some one better qualified for the task should fully satisfy himself of the value of belladonna; and, accordingly, Dr. Graefe, in 1856, published a paper in which he communicates the results of his experiments with mydriatics in the treatment of iritis, "the dangers of which," he says, "in general have materially diminished, since the introduction of a bold use of mydriatic remedies in the acute form."

[There are some people in whom great irritation follows the use of this drug. No matter what preparation is made use of, the distress to the patient is equally great.]

* Glycerine is the best vehicle for dissolving or thinning the extract, and possesses great advantage over water; it keeps moist for a long time when applied to the brow.

In such cases (of iritis), as well as where there is any intolerance of the drug, I am in the habit of taking away a small quantity of blood from the temple, by cupping, or by leeches applied either to the nose or lower eyelid; and should this fail, to paracentesis or division of the ciliary structures; especially the latter where there is diffuse cloudiness of the aqueous humor. Many of these to-be apprehended evils are in part, if not wholly due to some impurity of the drug; at other times to the careless preparation of its solution, such as dissolving the atropia in the strongest spirit of wine, or adding more spirit than is actually wanted to facilitate its perfect solution in the distilled water. Again, sometimes very strong solutions are employed, which, in my opinion, are highly objectionable. In no case have I found it necessary to exceed 4 grs. to the oz., and more frequently I employ a 2 gr. solution. Nothing, indeed, answers so well as the latter; and if this cannot be borne, we shall find it advantageous to apply over the brow an ointment of the sulphate of atropine, or the belladonna liniment of the British Pharmacopœia, both of which are efficient preparations. Their action is of course much slower, but in peculiar cases this is decidedly an advantage over the effects produced by the more rapidly acting solutions, which may certainly be classed among the abuses of this valuable agent in those peculiar forms of eye disease requiring the application of mydriatics to affect the movements of the iris.—*Journal of British Ophthalmology, October*, 1864, *p.* 54.

